# Rational antidepressant use

**DOI:** 10.1192/bjb.2018.33

**Published:** 2018-06

**Authors:** Daniel Dunleavy

**Affiliations:** Florida State University College of Social Work; email: djd09e@fsu.edu

In her contribution to the ‘Against the Stream’ series, Dr Moncrieff^1^ articulates the case for the drug-centred model of antidepressant action. She notes that antidepressants do not typically outperform placebo in well-designed studies (particularly in rare instances where an active placebo is used as a control^2^), have little clinical effect and can cause serious adverse effects. Having made the case that antidepressants are not ‘specific’ antidepressant agents, she makes some comments about their use in clinical practice. I would like to offer a few remarks about these issues, including some musings about what ‘rational antidepressant use’ might look like.

Modern psychiatric practice has seen the rise and fall of several promising antidepressant agents (the monoamine oxidase inhibitors, the tricyclic antidepressants and selective serotonin reuptake inhibitors (SSRIs)). Recent efforts include testing the possible antidepressant properties of ketamine. But are these efforts futile? Perhaps yes, perhaps no. A truly specific antidepressant drug (if one is ontologically possible) appears to be a pipedream, given current diagnostic limitations. Our categorisation of major depressive disorder is highly heterogeneous,^3^ creating a disjunctive category of cognitive, behavioural and biological symptoms that do not reliably cluster together. Even if any of our current drugs had specificity for ‘depression’, this would be extremely difficult to uncover in clinical practice or research settings. As a result, drug development will be prone to ideological, as opposed to scientific, revolutions.^4^

Should we therefore abandon antidepressants as a treatment modality? As long as we are honest with our patients about our current state of knowledge, I think not. Drug use has always been an integral part of human life,^5^ helping to alleviate life's various physical, emotional and existential pains. Antidepressants are no different in this respect. While researchers continue the search for a discrete condition called ‘depression’, drugs such as the SSRIs can be exploited for particular patient complaints. Antidepressants can cause emotional blunting, sedation, activation and decreased libido, among other things. Some have a proclivity towards one effect more than others. These effects can be exploited to relieve particular problems (e.g. sedation to alleviate insomnia, or emotional numbing to transcend an episode of intense anxiety or distress), without pretence towards a yet-to-be discovered condition. A rational provider would match a drug's effects to the patient's complaints, irrespective of diagnosis (or drug class); and would remain vigilant to the development of any adverse effects or deterioration of condition, start at the lowest recommended dose, and withdraw the patient from the drug as soon as possible. Psychosocial interventions can remain an important part of treatment, in many cases being the first treatment of choice. Antidepressants, like all drugs, are neither angels nor demons. They should be used selectively and thoughtfully, when used at all.
